# Discriminatory ability and prognostic evaluation of presepsin for sepsis-related acute respiratory distress syndrome

**DOI:** 10.1038/s41598-020-66121-7

**Published:** 2020-06-04

**Authors:** Jiangnan Zhao, Yan Tan, Li Wang, Yi Shi

**Affiliations:** 1 Department of Respiratory and Critical Medicine, Jinling Hospital, Medical School of Nanjing University, Nanjing, 210002 China; 2 Department of Respiratory Medicine, Nanjing First Hospital, Nanjing Medical University, Nanjing, 210002 China

**Keywords:** Biomarkers, Diseases, Medical research, Risk factors

## Abstract

Sepsis-related acute respiratory distress syndrome (ARDS) has worse clinical outcomes than non-sepsis-related ARDS. Presepsin is known to be elevated in sepsis, but little is known about its discriminatory ability and prognostic evaluation in patients with sepsis-related ARDS. This study was a multicenter prospective cohort study of 225 consecutive ARDS patients. Patients with sepsis-related ARDS had higher presepsin levels than patients with non-sepsis-related ARDS (P < 0.001). The area under the receiver operating characteristic (ROC) curve of presepsin (0.81) was significantly greater than that of PCT (0.62) in diagnosing sepsis-related ARDS (P = 0.001). Among patients with sepsis-related ARDS, presepsin levels were significantly higher in non-survivors than in survivors (P < 0.001). Presepsin was found to be an independent predictor of in-hospital mortality in sepsis-related ARDS. Based on ROC analysis, the addition of presepsin improved discrimination based on SOFA or APACHE II scores from 0.77 to 0.87 or 0.73 to 0.85 (all P < 0.05), respectively. The levels of plasma presepsin were positively correlated with disease severity, as determined by the SOFA score in the sepsis-related ARDS group (P < 0.001). Presepsin is a valuable biomarker for early stratification of sepsis-related ARDS. Higher plasma presepsin levels are associated with increased mortality in sepsis-related ARDS.

## Introduction

Acute respiratory distress syndrome (ARDS) is characterized by acute respiratory failure with severe hypoxemia and diffuse pulmonary infiltrates, which may occur after severe pulmonary and systemic injuries of septic and non-septic causes^1,2^. Despite the advances in supportive care strategies and significant efforts invested in research and clinical trials for ARDS, its mortality rate remains high, especially among patients with sepsis^3,4^. It may be favorable to characterize between septic and non-septic causes of ARDS because there is evidence to suggest that discrimination of these two subgroups might lead to improvements in future research and management of ARDS^5,6^. Early identification of sepsis-related or non-sepsis-related ARDS is challenging because patients with originally non-septic causes treated in intensive care units (ICUs) frequently acquire bacterial infection.

Biomarkers could contribute to the prompt identification of patients with sepsis who might benefit from quick and appropriate therapy. Among different biomarkers that have been suggested as sepsis biomarkers, presepsin appears quite promising in the early stages of the septic process^7–10^. Presepsin is a highly specific biomarker for diagnosing bacterial infections because it is produced in association with bacterial phagocytosis and cleavage of microorganisms by lysosomal enzymes^7,11^. Procalcitonin (PCT) has been used as a biomarker in sepsis, but has limited specificity and can be elevated in other predisposing scenarios of ARDS^10,12^. Presepsin appears to have superior capacity to diagnose sepsis compared to PCT^7,10,13^.

Previous clinical studies have confirmed that plasma presepsin levels are significantly increased in patients with sepsis, and are positively correlated with the severity of sepsis^7,9,13,14^. To date, no studies have queried whether presepsin differs between relevant subgroups in ARDS (i.e. sepsis and non-sepsis). Based on these premises, we designed a multicenter prospective study to determine whether presepsin levels differed between sepsis-related and non-sepsis-related ARDS and to investigate the clinical value of presepsin in early diagnosis and prognostic evaluation of sepsis-related ARDS.

## Results

### Study subjects

**Figure 1:**
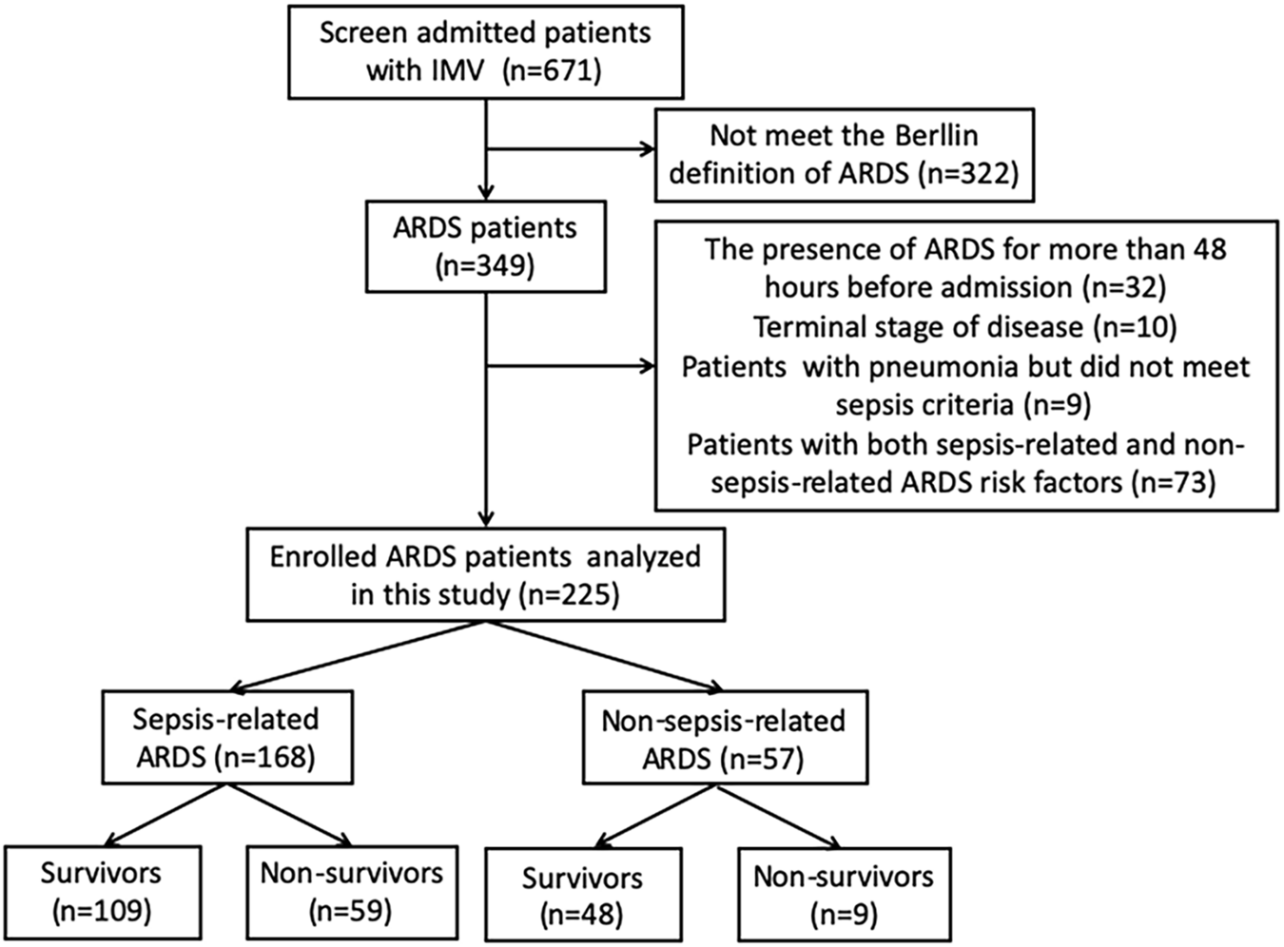
Flow chart of patient enrolment in the study. IMV: invasive mechanical ventilation, ARDS: acute respiratory distress syndrome.

Between September 2017 and August 2019, 225 ARDS patients and 100 healthy controls were enrolled in this study. Figure 1 illustrates the enrolment and follow-up of the study patients. A total of 671 patients with IMV were screened, of which 322 did not meet the ARDS criteria. After excluding 32 patients with the presence of ARDS for more than 48 hours, 10 patients with terminal-stage disease, 9 patients with pneumonia who did not meet the sepsis criteria, and 73 patients who exhibited both sepsis-related and non-sepsis-related ARDS risk factors, 225 patients remained for analysis and were followed up until discharge. The demographic characteristics, presepsin levels, PCT levels and associated infections in enrolled subjects are presented in Table 1. The demographic and clinical characteristics of the ARDS patients revealed a male predominance (70.2%). Regarding predisposing conditions, 168 (74.7%) patients had sepsis, and 57 (25.3%) had non-septic injuries. The associated infections in sepsis-related ARDS included pneumonia (70, 41.7%), intraabdominal infection (89, 53.0%), meningitis (3, 1.8%), pyelonephritis (4, 2.4%) and skin/soft tissue infection (2, 1.2%).

**Table 1:** Demographic characteristics, presepsin levels, PCT levels and associated infections in enrolled subjects.

	Controls	ARDS	Sepsis-related ARDS	Non-Sepsis- related ARDS
No	100	225	168	57
Age, years	51 (40–66)	58 (40–75)	58 (40–73)	59 (39–76)
Male sex	66 (66.0)	158 (70.2)	117 (69.6)	41 (71.9)
Presepsin (pg/ml)	52.5 (27.2–87.7)	470.3 (253.5–1135.0)	700.2 (342.1–1304.0)	262.0 (156.9–377.2)
PCT (ng/ml)	0.06 (0.04–0.08)	3.71 (1.26–11.20)	5.13 (1.21–15.49)	2.73 (1.33–4.04)
**Diagnosis**				
Pneumonia			70 (41.7)	
Intraabdominal infection			89 (53.0)	
Meningitis			3 (1.8)	
Pyelonephritis			4 (2.4)	
Skin/soft tissue infection			2 (1.2)	

Data are presented as median (25^th^–75^th^ percentile) or No (%). *ARDS* acute respiratory distress syndrome; *PCT* procalcitonin.

The characteristics of patients with sepsis-related ARDS and non-sepsis-related ARDS are shown in Table 2. No significant differences were found in age and sex between the two groups. Among patients with sepsis, 114 (67.9%) had septic shock, and 70 (41.7%) patients had pneumonia. Among patients with non-septic injuries, 30 (52.6%) had pancreatitis, 22 (38.6%) had aspiration, and 5 (8.8%) had trauma. Compared to patients with non-sepsis-related ARDS, those with sepsis-related ARDS were more likely to have diabetes (P = 0.006). Patients with sepsis-related ARDS also had significantly higher APACHE II scores (P < 0.001), higher SOFA scores (P = 0.014), greater WBC counts (P = 0.015), lower serum albumin levels (P = 0.016), and a greater frequency of vasopressor use at admission (P = 0.001) than patients with non-sepsis-related ARDS. Patients with sepsis-related ARDS had worse clinical outcomes than patients with non-sepsis-related ARDS, with significantly fewer ventilator-free days (P < 0.001), longer ICU days (P = 0.001), and higher in-hospital mortality rates (P = 0.007).

**Table 2:** Baseline characteristics of study groups.

Variables	ARDS (n = 225)	Sepsis-related ARDS (n = 168)	Non-Sepsis-related ARDS (n = 57)	P value
Age, years	58 (40–75)	58 (40–73)	59 (39–76)	0.499
Male sex	158 (70.2)	117 (69.6)	41 (71.9)	0.867
Current smokers	78 (34.7)	57 (33.9)	21 (36.8)	0.748
History of alcohol abuse	41 (18.2)	27 (16.1)	14 (24.6)	0.167
APACHE II score	25 (22–30)	26 (23–31)	22 (20–28)	<0.001
SOFA score	7 (6–10)	8 (6–10)	6 (5–9)	0.014
Predisposing conditions				
Sepsis-related	168 (74.7)	168 (100)	0	<0.001
Septic Shock	114 (50.7)	114 (67.9)	0	<0.001
Pneumonia	70 (31.1)	70 (41.7)	0	<0.001
Non-sepsis-related				
Aspiration	22 (9.8)	0	22 (38.6)	<0.001
Pancreatitis	30 (13.3)	0	30 (52.6)	<0.001
Trauma	5 (2.2)	0	5 (8.8)	<0.001
Comorbidities				
Hypertension	75 (33.3)	51 (30.4)	24 (42.1)	0.108
Diabetes	44 (19.6)	40 (23.8)	4 (7.0)	0.006
Coronary heart disease	21 (9.3)	15 (8.9)	6 (10.3)	0.794
Cerebrovascular disease	24 (10.7)	17 (10.1)	7 (12.3)	0.626
Chronic renal disease	40 (17.8)	28 (16.7)	12 (21.1)	0.548
COPD or asthma	23 (10.2)	16 (9.5)	7 (12.3)	0.614
Cancer	21 (9.3)	14 (8.3)	7 (12.3)	0.430
Laboratory values on ICU admission				
PCT, ng/ml	3.71 (1.26–11.2)	5.13 (1.21–15.49)	2.73 (1.33–4.04)	0.006
CRP, mg/L	98.0 (40.7–158.4)	103.5 (36.7–162.1)	86.8 (43–147.4)	0.485
WBC count, ×10^9^/L	8.8 (6.7–13.3)	9.1 (6.9–14.4)	8.1 (5.9–10.5)	0.015
Hematocrit, %	0.27 (0.24–0.33)	0.28 (0.25–0.34)	0.27 (0.22–0.31)	0.052
Platelet count, ×10^9^/L	199 (135–284)	194 (129–283)	218 (146–302)	0.269
Albumin, g/L	30.4 (27.3–34.3)	30.1 (26.8–33.5)	32 (28.3–35.4)	0.016
Bilirubin, μmol/L	13.8 (8.2–27.7)	14.1 (8.1–28.8)	12.8 (8.2–26.8)	0.601
Creatinine, μmol/L	77.7 (52.9–140.4)	77.8 (54.4–143.1)	77.1 (49.1–130.4)	0.662
Berlin categories				0.039
Mild	72 (32.0)	45 (26.8)	27 (47.4)	
Moderate	110 (48.9)	92 (54.8)	18 (31.6)	
Severe	43 (19.1)	31 (18.4)	12 (21.0)	
No. of organ failures	2 (1–3)	3 (2–4)	1 (1–2)	<0.001
Vasopressors use at admission	69 (30.7)	61 (36.3)	8 (14.0)	0.001
Clinical outcomes				
VFDs in 28 d	11 (3–15.5)	9 (2–14)	14 (11–21)	<0.001
Days in ICU of survivors	17 (10–28)	18 (11–33)	13 (8–20)	0.001
Hospital mortality	68 (30.2)	59 (35.1)	9 (15.8)	0.007

Data are presented as median (25^th^–75^th^ percentile) or No (%). *ARDS* acute respiratory distress syndrome; *SOFA* Sequential Organ Failure Assessment; *APACHE II* Acute Physiology and Chronic Health Evaluation II; *COPD* chronic obstructive pulmonary disease; *ICU* intensive care unit; *PCT* procalcitonin; *WBC* white blood cell; *CRP* C-reactive protein; No. of organ failures includes only non-pulmonary organ failures; VFDs: ventilator-free days.

### Discriminatory ability of presepsin

**Figure 2:**
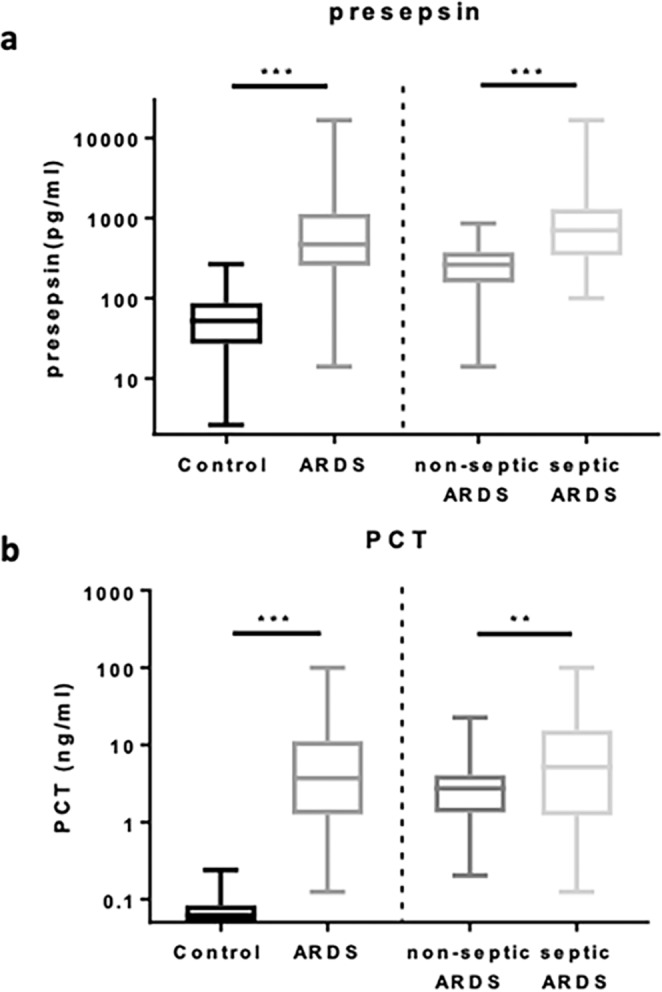
Plasma presepsin (**a**) and serum PCT (**b**) levels in controls and patients with ARDS. Data are presented as medians with 25^th^ and 75^th^ percentiles. ***P < 0.001 and **P < 0.01. PCT procalcitonin; ARDS: acute respiratory distress syndrome.

The levels of presepsin and PCT were significantly elevated in patients with ARDS compared to those in healthy controls (all P < 0.001) and were different between sepsis-related ARDS and non-sepsis-related ARDS (Fig. 2). Presepsin levels were markedly higher in patients with sepsis-related ARDS [700.2 pg/mL (342.1 pg/mL-1304.0 pg/mL)] than in patients with non-sepsis-related ARDS [262.0 pg/mL (156.9 pg/mL-377.2 pg/mL)] (P < 0.001). Patients with sepsis-related ARDS had significantly higher PCT levels [5.13 ng/mL (1.21 ng/mL-15.49 ng/mL)] than patients with non-sepsis-related ARDS [2.73 ng/mL (1.33 ng/mL-4.04 ng/mL)] (P = 0.006); meanwhile, there was a clear overlap between the two groups regarding serum PCT levels. Compared with those in the healthy control group, presepsin and PCT levels were also obviously higher in patients with non-sepsis-related ARDS.

**Figure 3:**
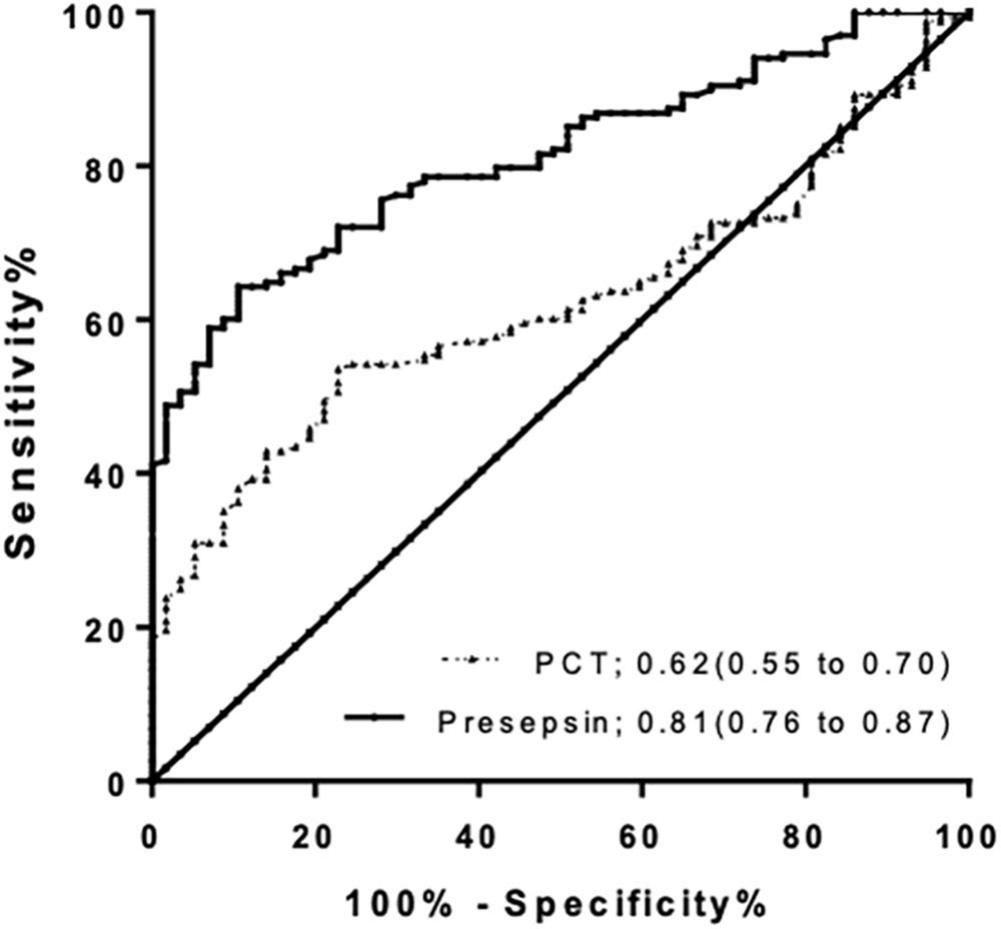
ROCs of presepsin and PCT for the diagnosis of sepsis-related ARDS. Areas under the ROCs: presepsin 0.81(95% CI 0.76 to 0.87), P < 0.001; and PCT 0.62 (95% CI 0.55 to 0.70), P = 0.006. ROC: receiver operating characteristic; PCT procalcitonin; ARDS: acute respiratory distress syndrome.

The ROC curves of presepsin and PCT for diagnosing sepsis-related ARDS are shown in Fig. 3. The AUC of presepsin [0.81 (95% CI 0.76–0.87)] was significantly higher than that of PCT [0.62 (0.55–0.70)] (P < 0.01). Using the presepsin cutoff value of 454.3 pg/ml for predicting sepsis-related ARDS, specificity, sensitivity, Youden’s index, PPV, NPV, + LR and −LR were found to be 89.5%, 64.3%, 0.538, 94.7%, 45.9%, 6.12, and 0.40, respectively (Table 3).

**Table 3:** Performance of presepsin for predicting sepsis-related ARDS and in-hospital mortality in sepsis-related ARDS.

	Sepsis-related ARDS	In-hospital mortality
Cutoff	454.3 pg/ml	940.8 pg/ml
Specificity	89.5%	73.4%
Sensitivity	64.3%	57.6%
Youden’s index	0.538	0.310
PPV	94.7%	54.0%
NPV	45.9%	76.2%
+LR	6.12	2.17
−LR	0.40	0.58

ARDS acute respiratory distress syndrome; PPV positive predictive value; NPV negative predictive value; +LR positive likelihood ratio; −LR negative likelihood ratio.

### Prognostic evaluation of presepsin

**Figure 4:**
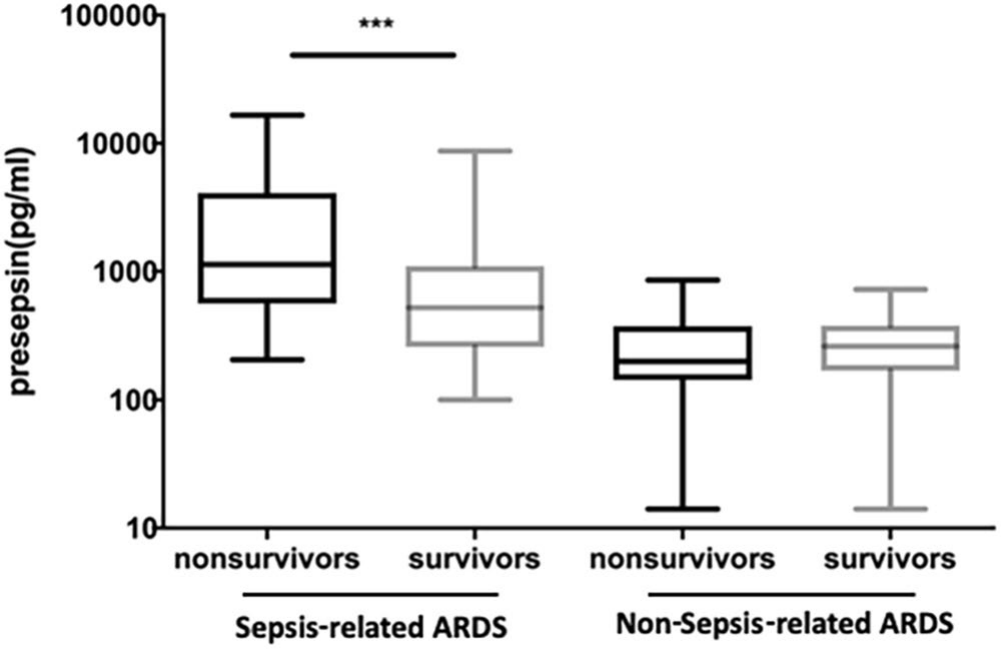
Presepsin levels at admission in survivors and non-survivors of patients with sepsis-related and non-sepsis-related ARDS. Data are presented as medians with 25^th^ and 75^th^ percentiles. ***P < 0.001. ARDS: acute respiratory distress syndrome.

After multivariate logistic regression, presepsin levels were not independently associated with mortality in total ARDS patients. Because of higher presepsin levels in sepsis-related ARDS, we performed analysis stratified by type of etiologies. In patients with sepsis-related ARDS, the median levels of presepsin were significantly higher in non-survivors than in survivors [1136 pg/mL (704.9 pg/mL to 5476.0 pg/mL) vs. 470.3 pg/mL (259.6 pg/mL to 993.8 pg/mL); P < 0.001] (Fig. 4). This difference was not present in non-sepsis-related ARDS (P = 0.891). In multivariate regression analysis, age (OR 1.08; 95% CI 1.01–1.39), presepsin (OR 1.51; 95% CI 1.05–2.16), SOFA score (OR 1.78; 95% CI 1.18–2.68), APACHE II score (OR 1.58; 95% CI 1.06–2.35), and number of organ failures (OR 2.01; 95% CI 1.12–3.56) were found to be independent predictors of in-hospital mortality in patients with sepsis-related ARDS (Table 4). We also analyzed the independent predictor for fatal outcome in non-sepsis-related ARDS. Using logistic regression analysis, age (OR 1.05; 95% CI 1.02–1.09), APACHE II score (OR 1.15; 95% CI 1.02–1.30), number of organ failures (OR 2.49; 95% CI 1.32–5.17) and PaO_2_/FiO_2_ ratio (OR 1.84; 95% CI 1.18–3.58) were the independent risk factors for in-hospital mortality among non-sepsis-related ARDS patients (supplementary Table S1).

**Table 4:** Performance of multivariate-logistic regression for predicting in-hospital mortality in patients with sepsis-related acute respiratory distress syndrome.

Variables	OR	95% confidence interval		P value
		Lower limit	Upper limit	
Age	1.08	1.01	1.39	<0.001
Presepsin	1.51	1.05	2.16	0.027
SOFA score	1.78	1.18	2.68	0.008
APACHE II score	1.58	1.06	2.35	0.026
No. of organ failures	2.01	1.12	3.56	0.019

*SOFA* Sequential Organ Failure Assessment; *APACHE II* Acute Physiology and Chronic Health Evaluation II; No. of organ failures includes only non-pulmonary organ failures; *OR* odds ratio.

**Figure 5:**
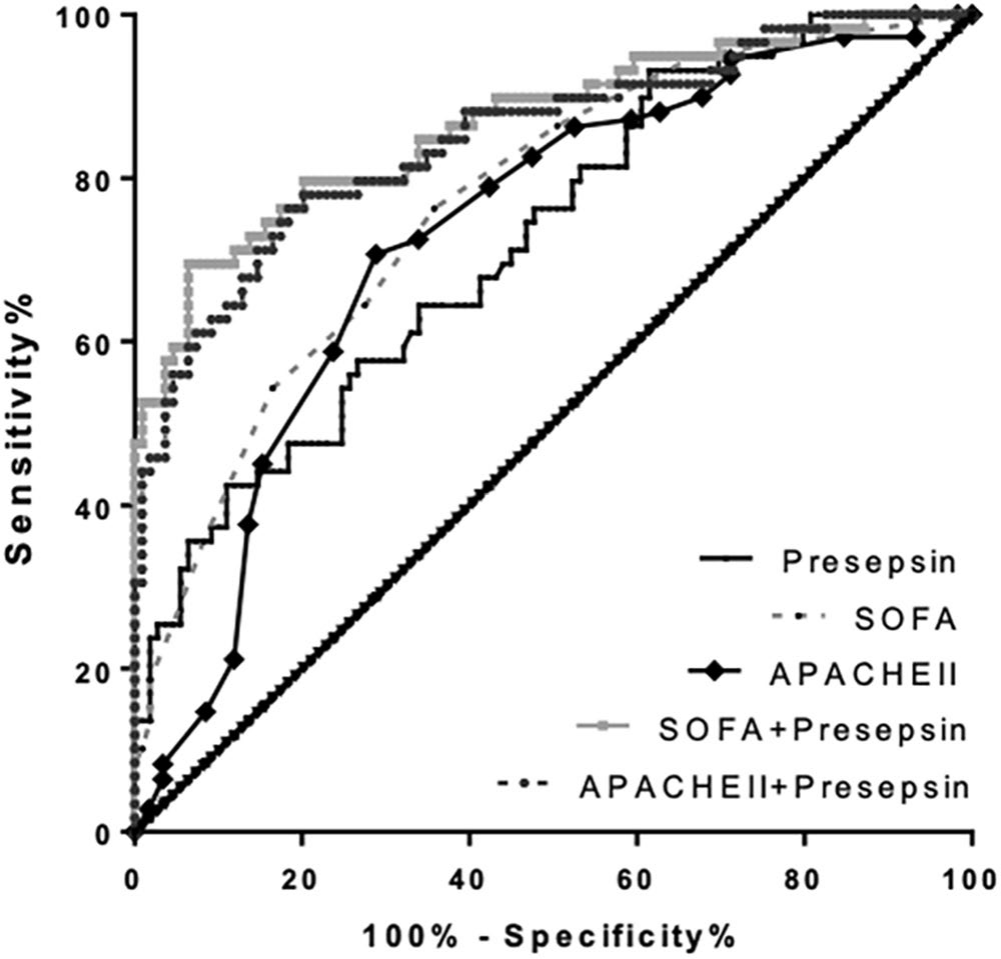
ROCs depicting improvement in predicting in-hospital mortality of patients with sepsis-related ARDS based on the SOFA and APACHE II scores with the addition of presepsin levels to the model. The AUC increased from 0.77 to 0.87 (P < 0.05) and 0.73 to 0.85 (P < 0.05), respectively. ROC: receiver operating characteristic; ARDS: Acute respiratory distress syndrome; SOFA: Sequential Organ Failure Assessment; APACHE II: Acute Physiology and Chronic Health Evaluation II; AUC: areas under the curves.

The AUC of presepsin for predicting in-hospital mortality in patients with sepsis-related ARDS was 0.72, while the AUC of SOFA and APACHE II scores were 0.77 and 0.73, respectively (Fig. 5). Using a presepsin cutoff value of 940.8 pg/ml for predicting hospital mortality in sepsis-related ARDS, the specificity was 73.4%, the sensitivity was 57.6%, the Youden’s index was 0.310, the PPV was 54.0%, the NPV was 76.2%, the LR + was 2.17, and the LR- was 0.58 (Table 3). The AUCs of presepsin in combination with the SOFA and APACHE II scores were 0.87 and 0.85, respectively, which were significantly higher than that of presepsin, SOFA score or APACHE II score alone (all P < 0.05). The detailed results are illustrated in supplementary Table S2.

### Correlation of presepsin with SOFA

**Figure 6:**
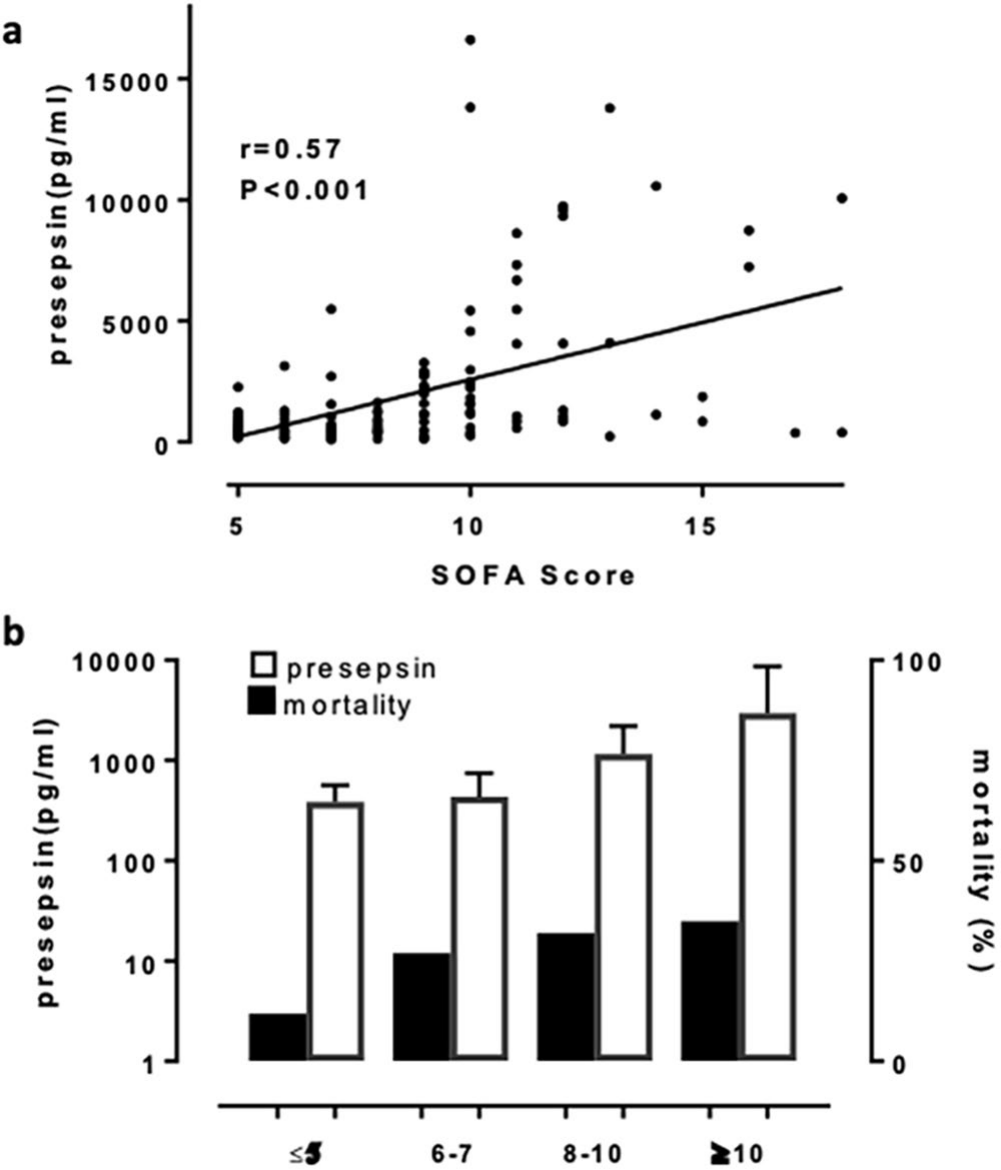
In patients with sepsis-related ARDS, presepsin levels were associated with the SOFA score and mortality. (**a**) Linear regression analysis and 95% CI with the SOFA score as the dependent variable and plasma presepsin levels. (**b**) Mortality rates in relation to plasma presepsin levels and the SOFA score. Bars indicate medians and interquartile ranges for presepsin. ARDS: Acute respiratory distress syndrome; SOFA: Sequential Organ Failure Assessment; CI: confidence interval.

The SOFA score at ICU admission is a good indicator of prognosis and appears to be a particularly useful predictor of outcome^15^. We noted a significant positive correlation between the SOFA score and presepsin levels with a correlation coefficient (r) of 0.57 (P < 0.001; Fig. 6a). To demonstrate that plasma presepsin levels are associated not only with disease severity but also with mortality, the patients were classified into four groups with increasing quartiles of the SOFA score. For each group, the mortality rate and plasma presepsin levels were calculated. As shown in Fig. 6b, presepsin levels were significantly correlated with in-hospital mortality (P < 0.001).

## Discussion

ARDS onset is often rapid and progressive, resulting from either septic or non-septic insults^16^. Sepsis as the most commonly encountered cause of ARDS is generally associated with higher mortality than other risk factors^3,17–19^. Our study demonstrated that sepsis- and non-sepsis- related ARDS present differently in terms of clinical characteristics, with higher severity of illness and worse clinical outcomes in sepsis-related ARDS. Response to treatment and clinical outcomes differ on the basis of sub-phenotype^20^. Early recognition of individual patients with sepsis-related ARDS is recommended during ICU treatment in order to establish targeted therapies.

However, the diagnosis and stratification of ARDS are based on clinical definitions that lack both sensitivity and specificity. Clinical symptoms and signs can be misleading, especially in patients with variable clinical characteristics or several comorbidities; thus, early diagnosis of sepsis-related ARDS is not straightforward. Recent interest has focused on blood biomarkers, which might capture aspects of pathophysiology that are not otherwise well captured in clinical data, and generally contribute more prominently to the sub-phenotype determination in clinical work^21^.

Presepsin, a 13-kDa fragment of sCD14, is released in the plasma as a consequence of cellular phagocytosis after bacterial infection and appears to be a novel biomarker of sepsis^22^. It has been used for early diagnosis, risk stratification and prognostic evaluation of sepsis in recent years^7,8,10,13^. However, no studies have tested the possible value of presepsin in patients with sepsis who have developed ARDS. Thus, we designed a multicenter prospective study to validate the diagnostic and prognostic role of presepsin in sepsis-related ARDS; and compare it with the clinical value of PCT.

As our results show, compared to that in healthy controls, presepsin increases considerably in patients with ARDS. Subpopulation analysis showed a significant correlation between presepsin levels and the different populations in the ARDS group (sepsis-related ARDS and non-sepsis-related ARDS). Presepsin was invariably elevated in patients with sepsis-related and non-sepsis-related ARDS. Patients with sepsis-related ARDS had notably higher plasma presepsin levels than patients with non-sepsis-related ARDS. The difference was present in the early phase of evolving ARDS, thereby allowing the discrimination between sepsis-related and non-sepsis-related ARDS before the results of microbiological testing are generally available. Presepsin seems to be a diagnostic tool for differentiating between septic and non-septic underlying disease in early ARDS.

Although serum PCT has been used as a biomarker in the diagnosis of sepsis, PCT is also increased in other risk conditions of ARDS, such as multiple trauma, extensive burns, pancreatitis, organ transplantation, and major surgery^12^. In our setting, PCT was elevated in both patient groups with a substantial overlap between patients with sepsis-related and non-sepsis-related ARDS patients. The median levels of PCT were significantly more elevated in sepsis-related ARDS, while ROC curve analysis showed that PCT concentrations had less valuable diagnostic capacity for sepsis-related ARDS than presepsin. Therefore, PCT shows limited value for distinguishing sepsis from other etiologies in patients with ARDS.

Higher levels of plasma presepsin were associated with worse clinical outcomes in patients with sepsis^7,23,24^. In our patients with sepsis-related ARDS, presepsin concentrations were higher in decedents than in survivors. Analysis of mortality in sepsis-related ARDS showed that a significant correlation between presepsin levels and mortality at early stages. Measurements of presepsin levels revealed valuable prognostic capacity to predict all-cause in-hospital mortality. After adjustments for other clinical variables, presepsin retained acceptable prognostic value for in-hospital mortality. The SOFA score, a good indicator of prognosis evaluating six different organ systems and addressing diverse clinical parameters, predicts the severity and mortality of critically ill patients with high accuracy^15^. A significant correlation was found between presepsin levels and the SOFA score in patients with sepsis-related ARDS. It is enticing that the prognostic accuracy of presepsin appeared to be similar to that of the SOFA score for early mortality in patients with sepsis-related ARDS. However, we are not suggesting the application of presepsin as the sole marker for predicting mortality, but merely emphasizing its association with mortality and the associated implications for potentially contributing to risk stratification in combination with other clinical tools (such as risk-prediction scores).

To the best of our knowledge, the results presented herein are the first with regard to the discriminative value and prognostic capacity of presepsin for sepsis-related ARDS. Presepsin may add to diagnostic accuracy and facilitate early recognition of patients with sepsis-related ARDS who are likely to benefit from promptly appropriate broad-spectrum antibiotics. Our results also pointed out the possible prognostic role of presepsin in promptly identifying high-risk patients with sepsis-related ARDS and predicting in-hospital mortality. The application of presepsin in ARDS sub-phenotypes (sepsis-related and non-sepsis-related) might be a useful tool to stratify patients in future clinical research trials, which might be advantageous for future differential treatments.

A major strength of this study is that it was conducted in two large, well-defined centers and four ICUs. All data were collected prospectively to avoid recall biases. Nevertheless, several limitations should be acknowledged. First, the number of patients was relatively small; subsequent studies with large-scale and independent cohorts should confirm and validate the clinical indications for presepsin in sepsis-related ARDS. Second, there were no sepsis and non-sepsis controls without ARDS in our study. Future research will be performed by addition of sepsis and non-sepsis controls without ARDS, which would help gain some insight as to the relevance of presepsin to sepsis-related ARDS. Third, the study design limited patients to those who required IMV, thereby not generalizing patients who met the ARDS criteria but only received non-IMV. Fourth, only one measurement of presepsin was available. Determination of the presepsin kinetics would be helpful and rewarding during the management of disease. Finally, we were not able to perform the commonly accepted Cox regression because of limitations in our data.

Our study is the first to assess presepsin in ARDS of sepsis or non-sepsis etiology. Elevated presepsin levels, when measured in the early course of ARDS, provided excellent discrimination for the clinical diagnosis of sepsis-related ARDS compared to PCT; and were also associated with an increased risk of mortality among patients with ARDS due to sepsis injury. Although further confirmatory studies are warranted, presepsin seems to be a promising biomarker for early differentiation of septic underlying disease in ARDS and evaluation of prognosis in sepsis-related ARDS.

## Methods

### Study design and patient inclusion

Study patients were recruited from four ICUs (two respiratory ICUs, one medical ICU, one emergency ICU) in Jinling Hospital and Nanjing First Hospital between September 2017 and August 2019. All the consecutively admitted patients were screened for enrolment if they were undergoing invasive mechanical ventilation (IMV), had a PaO_2_/FiO_2_ ratio of 300 or less, and exhibited bilateral infiltrates on chest radiography that were present concurrently. Enrolled patients were further evaluated on the basis of the Berlin criteria of ARDS^25^. The general ventilatory and medical management were presented in supplementary material S1.

The exclusion criteria were as follows: <18 years old, the presence of ARDS for more than 48 hours before admission, the presence of initial antibiotic treatment before enrolment, terminal stage of disease (malignant cancer of any type, acquired immunodeficiency syndrome, end-stage liver or renal disease), and refusal of consent to inclusion by the patient or relatives.

Paired blood samples were obtained simultaneously from age- and sex-matched healthy volunteers. Participants of legally authorized surrogates provided written informed consent. All experiments were performed in accordance with relevant guidelines and regulations. This study was approved by the Jinling Hospital and Nanjing First Hospital Ethics Committee (Approval Number: JLYY: 2013021).

### Data collection and definitions

Patient demographic and baseline clinical characteristics were recorded on study enrolment. Vital signs, laboratory values, imaging scans and other data in the first 24 hours of ARDS onset were collected. The Sequential Organ Failure Assessment (SOFA) and Acute Physiology and Chronic Health Evaluation II (APACHE II) scores were calculated to assess disease severity^15,26^.

Patients with predisposing conditions for ARDS (see electronic supplementary material S2, for details of the inclusion criteria), including sepsis, sepsis shock, pneumonia, aspiration, pancreatitis and trauma, were further divided into sepsis-related ARDS and non-sepsis-related ARDS groups. According to previous literature^4^, sepsis-related ARDS were identified as ARDS developing in patients with sepsis and non-sepsis-related ARDS as that developing after non-septic injuries, such as aspiration, acute pancreatitis and trauma. We considered ARDS developing in patients with pneumonia who fulfilled the sepsis criteria as sepsis-related ARDS and excluded patients with pneumonia who did not meet the sepsis criteria. Patients with both sepsis-related and non-sepsis-related risks for ARDS were excluded from analysis.

Patients were followed up until death or discharge from the ICU. We used all-cause in-hospital mortality as the major clinical outcome. Patients with sepsis-related ARDS were classified into surviving and non-surviving groups according to in-hospital survival. Other short-term outcomes were measured in ventilator-free days and length of ICU stay.

### Biomarkers measurement

Venous blood samples were obtained as soon as possible within 24 hours following enrolment in the study to measure presepsin and to determine other clinical parameters. Presepsin was measured in plasma. The plasma presepsin measurements were carried out blindly in duplicate using enzyme-linked immuno-sorbent assay (ELISA) kits (BioVision. Inc., USA). Blood samples were collected in tubes containing ethylenediamine tetraacetate and centrifuged at 2000 rpm for 15 minutes. Plasma was separated and stored at −80 °C. PCT was measured in serum samples using ELISA kits (Cusabio, Wuhan, P.R. China). After coagulation at 4 °C, serum was separated by centrifugation at 2000 rpm for 15 minutes and immediately stored at −80 °C until measurement. Concordance of duplicates in ELISA testing was well.

### Statistical analysis

All continuous variables were non-normally distributed and thus expressed as the median (interquartile range [IQR]). Differences between groups were evaluated using either the nonparametric Mann-Whitney U test for two groups or the Kruskal-Wallis analysis of variance for more than two groups. Categorical variables presented as frequencies and percentages were compared using Pearson’s *X*^2^ or Fisher’s exact test. Receiver-operating characteristic (ROC) curves were constructed, and the areas under the ROC curves (AUCs) were determined. Optimal thresholds were determined on the basis of ROC curves analysis, and prognostic parameters [sensitivity, specificity, Youden’s index, positive predictive value (PPV), negative predictive value (NPV) as well as positive likelihood ratio (+LR) and negative likelihood ratio (−LR)] were also calculated. Binary logistic analysis was applied to create a new score combining two factors predicting risk. Diagnostic AUCs were compared by Z-test. The test standard was Z_0.05_ = 1.96 and Z_0.01_ = 2.58. Multivariate logistic regression analysis was applied to determine the independent predictors of in-hospital mortality in sepsis-related ARDS. The odds ratios (ORs), 95% confidence intervals (CIs), and P values for individual variables were obtained. Correlations were assessed using linear regression or Spearman analysis. All data were analyzed using SPSS 24.0 software, and P < 0.05 was considered statistically significant.

### Ethics approval and consent to participate

Informed consent was obtained from patients’ legal representatives. All research was performed in accordance with relevant guidelines. The protocol was approved by the ethics committee of Jinling Hospital and Nanjing First Hospital (Approval Number: JLYY: 2013021).

## Supplementary information

Supplementary Information.

## Data Availability

All data generated or analyzed during this study are included in this published article.
