# Supplementary material for: Discriminatory ability and prognostic evaluation of presepsin for sepsis-related acute respiratory distress syndrome
Source: Sci Rep. 2020 Jun 4;10:9114. doi: 10.1038/s41598-020-66121-7 (PMC7272415; doi:10.1038/s41598-020-66121-7)
Supplement: Supplementary file 1 — Supplementary Information. [file 41598_2020_66121_MOESM1_ESM.docx]

**Supplementary information**

**Discriminatory ability and prognostic evaluation of presepsin for sepsis-related acute respiratory distress syndrome**

Jiangnan Zhao^1^, Yan Tan^2^, Li Wang^2^, Yi Shi^1^*

1Department of Respiratory and Critical Medicine, Jinling Hospital, Medical School of Nanjing University, Nanjing, 210002, China.

2Department of Respiratory Medicine, Nanjing First Hospital, Nanjing Medical

University, Nanjing, 210002, China.

First authors: Jiangnan Zhao

*Corresponding author: Yi Shi, PhD

Email: yishi201607@163.com

Institution of Yi Shi: Department of Respiratory and Critical Medicine, Jinling Hospital, Medical School of Nanjing University;

Address: No. 305 Zhongshan East Road, Nanjing, 210002, China

Work telephone number: 13951708712; Work fax number: 025-80860148;

**Supplementary Material 1**

General management

Patients were ventilated with protective ventilation using low tidal volume 4-8ml/kg of predicted body weight plus moderate to high levels of positive-end expiratory pressure (PEEP) for volume-controlled or pressure-controlled ventilation ^1^. For male patients, the predicted body weight was calculated as equal to 50+0.91(centimeters of height-152.4); for female, as equal to 45.5+0.91(centimeters of height-152.4). Ventilatory adequacy was monitored by arterial blood gas measurements, with settings changed as needed. The FiO_2_ was adjusted to maintain pulse oximetry＞90% or PaO_2_＞60 mmHg. Avoid raising the peak inspiratory pressure over 35cm H_2_O. The general medical management including fluid replacement, the use of antibiotics, corticosteroids in some selected patients, vasopressor agents, sedation and paralysis was directed by the intensivists-in- charge.

**Supplementary Material 2**

Inclusion criteria of ARDS study

Sepsis: A known or suspected source of systemic infection plus at least two of the followings: a) heart rate > 90 beats per minute; b) body temperature > 38℃ or < 36℃; c) respiratory rate > 20 breaths/min or PaCO_2_ < 32mmHg; d) WBC > 12×10^9^cells/L or < 4×10^9^cells/L. The infections in our study were clinically established on the basis of clinical features, laboratory findings, microbiologic tests and imaging studies according to the criteria of the International Sepsis Forum Consensus Conference on Definitions of Infection^2^. The diagnosis of community-acquired pneumonia was mainly based on a new infiltrate plus at least one recently acquired respiratory symptom (cough, sputum production, dyspnea, tachypnea, pleuritic pain) or sign (auscultatory findings of abnormal breath sounds and rales). Intra-abdominal infections comprised the following diseases in our study: the diagnosis of peritonitis was based on clinical findings including abdominal pain, tenderness to palpation, and peritoneal signs such as rigidity or rebound tenderness, which were supported by radiographic findings, such as free air under the diaphragm or localized fluid collection visualized by computed tomography with a compatible clinical illness; the diagnosis of biliary tract infection was on the basis of clinical evidence of biliary tract infection with surgical or radiographic evidence of supportive complications; typhlitis was diagnosed according to a compatible clinical presentation with radiographic evidence of bowel wall edema and/or gas and/or hemorrhagic necrosis within the bowel wall of the cecum; and the diagnosis of pyelonephritis was based on clinical features (temperature>38°C, urgency, localized pain or tenderness at involved site), and pyuria, hematuria, and radiographic evidence of infection. Skin and soft-tissue infections (cellulitis) were suggested by the presence of a rapidly expanding erythema, local tenderness, pain, swelling, lymphangitis, and lymphadenopathy, which is frequently accompanied by systemic signs and symptoms including malaise, temperature>38.0°C, and chills. Determination of bacterial meningitis was based on compatible clinical features, and cerebrospinal fluid findings (cerebrospinal fluid leukocyte count >1×10^9^cells/L)^3^.

Septic shock: Fulfill requirements for sepsis plus one of the following: a) systolic blood pressure (SBP) < 90 mmHg or a reduction of ≥ 40 mmHg from baseline for ≥ 30 mins, unresponsive to 500 mL of fluid resuscitation; b) need for vasopressors to maintain SBP ≥ 90 mmHg or within 40 mmHg of baseline.

Pneumonia: Fulfill two or more of the following: a) new airspace opacity on chest radiograph; b) temperature>38.3°C or<36.0°C, WBC>12×10^9^cells/L or <4×10^9^cells/L, c) positive microbiological culture.

Aspiration: Witnessed or documented aspiration event or the retrieval of gastric contents from the oropharynx, endotracheal tube, or bronchial tree.

Acute pancreatitis: An acute inflammatory process of the pancreas that may also involve peripancreatic tissues and/or remote organ systems, requiring two of the following three features: a) abdominal pain characteristic of acute pancreatitis; b) serum amylase and/or lipase ≥ 3 times the upper limit of normal; c) characteristic findings of acute pancreatitis on CT scan.

Trauma: Multiple fractures and/or pulmonary contusions. Multiple fractures are defined as fractures of two long bones; an unstable pelvic fracture; or one major long bone and a pelvic fracture. Pulmonary contusion is defined as airspace opacity on chest radiograph within 8 hours of admission to the emergency room and evidence of blunt trauma to the chest, for example, fractured ribs or ecchymosis overlying airspace opacity.

References

1 Acute Respiratory Distress Syndrome, N. *et al.* Ventilation with lower tidal volumes as compared with traditional tidal volumes for acute lung injury and the acute respiratory distress syndrome. *N Engl J Med* **342**, 1301-1308, doi:10.1056/NEJM200005043421801 (2000).

2 Calandra, T., Cohen, J. & International Sepsis Forum Definition of Infection in the, I. C. U. C. C. The international sepsis forum consensus conference on definitions of infection in the intensive care unit. *Crit Care Med* **33**, 1538-1548, doi:10.1097/01.ccm.0000168253.91200.83 (2005).

3 Tunkel, A. R. *et al.* Practice guidelines for the management of bacterial meningitis. *Clin Infect Dis* **39**, 1267-1284, doi:10.1086/425368 (2004).

**Supplementary Table S1.** Multivariate logistic regression analysis of clinical variables associated with in-hospital mortality in patients with non-sepsis-related ARDS

| Variables | OR | 95% confidence interval | | | P value |
| --- | --- | --- | --- | --- | --- |
|  |  | Lower limit | | Upper limit |  |
| Age | 1.05 | 1.02 | 1.09 | | 0.003 |
| APACHE II score | 1.15 | 1.02 | 1.30) | | 0.025 |
| No. of organ failures | 2.49 | 1.32 | 5.17 | | 0.035 |
| PaO_2_/FiO_2_ ratio | 1.84 | 1.18 | 3.58 | | 0.008 |

*ARDS* Acute respiratory distress syndrome; *OR* odds ratio, *APACHE II* Acute Physiology and Chronic Health Evaluation II; No. of organ failures includes only non-pulmonary organ failures

**Supplementary Table S2.** Areas under the curves of various parameters for predicting in-hospital mortality in patients with sepsis-related acute respiratory distress syndrome

| Variables | AUC | 95% confidence interval | | P value |
| --- | --- | --- | --- | --- |
|  |  | Lower limit | Upper limit |  |
| Presepsin | 0.72 | 0.65 | 0.80 | <0.001 |
| SOFA score | 0.77 | 0.70 | 0.84 | <0.001 |
| APACHE II score | 0.73 | 0.65 | 0.81 | <0.001 |
| Presepsin + SOFA score | 0.87 | 0.81 | 0.93 | <0.001 |
| Presepsin + APACHE II score | 0.85 | 0.78 | 0.91 | <0.001 |

*AUC* areas under the curves; *SOFA* Sequential Organ Failure Assessment; *APACHE II* Acute Physiology and Chronic Health Evaluation II
